# Immunostimulatory and antioxidant activities of the selenized polysaccharide from edible *Grifola frondosa*

**DOI:** 10.1002/fsn3.2764

**Published:** 2022-02-02

**Authors:** Qian Li, Linfei Zhu, Xingpu Qi, Ting Zhou, Yonglian Li, Mingjie Cai, Yuting Yan, Jian‐Ya Qian, Daxin Peng

**Affiliations:** ^1^ College of Veterinary Medicine Yangzhou University Yangzhou China; ^2^ School of Food Science and Engineering Yangzhou University Yangzhou China; ^3^ College of Food Science and Technology Jiangsu Agri‐animal Husbandry Vocational College Taizhou China; ^4^ School of Agricultural Equipment Engineering Jiangsu University Zhenjiang China

**Keywords:** antioxidant activity, *Grifola frondosa*, lymphocyte proliferation, polysaccharide, selenylation modification

## Abstract

*Grifola frondosa* polysaccharide (GFP2) was extracted and purified by anion‐exchange chromatography. A selenized *G. frondosa* polysaccharide, SeGFP2, was modified in selenylation by nitric acid–sodium selenite (HNO_3_‐Na_2_SeO_3_) method. Structural features were investigated, and the lymphocyte proliferation and antioxidant activities were compared taking GFP2 as control. SeGFP2 with a molecular weight of 2.12 × 10^4^ Da was composed of mannose, glucose, and galactose with a ratio of 3.5:11.8:1.0. A typical absorption of selenium ester was observed in SeGFP2 molecule. SeGFP2 was proposed as a branched polysaccharide, which consisted of 1,3‐D‐Glc*p*, 1,6‐D‐Glc*p*, 1,4,6‐D‐Gal*p,* and 1,3,6‐D‐Man*p*. SeGFP2 showed a linear filamentous structure with some branches. SeGFP2 could significantly promote T‐ or B‐lymphocyte proliferation and the enhancement was higher than GFP2. The in vitro antioxidant activities of SeGFP2 were more potent than GFP2. These present data suggested that selenylation could significantly improve the lymphocyte proliferation and in vitro antioxidant activities of GFP2.

## Graphical abstract

A novel selenized polysaccharide (SeGFP2) from *G. frondosa* was prepared. Structural features were elucidated by GC‐MS, methylation, HPSEC‐MALLS‐RI, etc. SeGFP2 with low molecule mass exhibited stronger immunostimulatory ability. A selenized *G. frondosa* polysaccharide, SeGFP2, was modified in selenylation by nitric acid–sodium selenite (HNO3‐Na2SeO3) method. Structural features were investigated, and the lymphocyte proliferation and antioxidant activities were compared taking GFP2 as control.

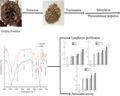

## 1 INTRODUCTION

Selenium (Se) is a necessary microelement which is important for the development and maintenance of organisms. Se has been found to exhibit various biological effects, such as immunomodulatory, antioxidant, antitumor, and hypoglycemic and hypolipidemic effects (Hartikainen, 2005). Se is a key component of Se‐dependent enzymes, such as glutathione peroxidase (GSH‐Px) and thioredoxin reductase (TrxR). Se deficiency could cause many diseases in humans, such as immune dysfunction, cancer, and hypothyroidism (Rzymski et al., 2017). As a major source of Se, selenium supplement is imperative for humans, especially in Se deficiency regions. It shall be noted that organic Se shows a higher bioavailability, as it can be readily absorbed in human digestive tracts and also has higher threshold for the toxicity compared with inorganic Se. In addition, biotransformation and chemical synthesis of Se‐proteins or Se‐polysaccharides have been widely used to prepare organic Se compounds, and attracted tremendous attention of researchers and consumers recently (Hou et al., 2016; Zhang, Gao, et al., 2021).

Se‐polysaccharides could exert the efficacy of both polysaccharide and Se, and the biological activity is usually higher than that of polysaccharide or Se (Zhang, Gao, et al., 2021). Generally, Se‐polysaccharides from biotransformation method mainly exist in plants, mushrooms, and microorganisms. The quality of the Se‐polysaccharides is influenced by both the area and season. The Se content and selenium translation rate of the Se‐polysaccharides from biotransformation method are relatively lower than chemical selenylation, even in a high selenium area or liquid medium (Zhang, Lu, et al., 2016). Recently, it has been reported that the chemical selenylation of Se‐polysaccharides involved nitric acid–sodium selenite (HNO_3_‐Na_2_SeO_3_), glacial acetic acid–selenous acid (CH_3_COOH‐H_2_SeO_3_), glacial acetic acid–sodium selenite (CH_3_COOH‐Na_2_SeO_3_), and selenium oxychloride (SeCl_2_O) method (Gao et al., 2016).

*Grifola frondosa*, an edible mushroom assigned to the Polyporaceae family, has been found to have diverse medicinal values. Due to the existence of bioactive polysaccharides, *G. frondosa* has become increasingly popular and widely cultivated in China (Klaus et al., 2015). These polysaccharides have been reported to possess potential biological effects, including immunostimulatory, antioxidant, antitumor, antidiabetic, and antihypertensive activities (Li et al., 2017; Meng et al., 2017). Bioactive polysaccharides extracted from the fruit bodies or mycelia of *G. frondosa* have attracted the most attention due to their diverse structure and potentially significant pharmacological activities.

In this study, the *G. frondosa* polysaccharides were extracted and purified by anion‐exchange chromatography, and modified in selenylation by HNO_3_‐Na_2_SeO_3_ method for the first time. The lymphocyte proliferation and antioxidant activities of selenized *G. frondosa* polysaccharides (SeGFP2) were evaluated taking *G. frondosa* polysaccharides as control. The structural features of SeGFP2 were explored by Fourier transform‐infrared (FT‐IR) spectrometry, monosaccharide components analysis, methylation, gas chromatography–mass spectrometry (GC‐MS), high‐performance size exclusion chromatography–multiangle laser light scattering–refractive index detector (HPSEC‐MALLS‐RI), Congo red spectrophotometric analysis, circular dichroism (CD), and atomic force microscope (AFM). Overall, this information would be helpful for the development of novel functional foods or drugs using the Se‐polysaccharide as an ingredient.

## 2 MATERIALS AND METHODS

### 2.1 Materials

The fruit bodies of *G. frondosa* (Bacterial number: Qing gray 151) were harvested in Qingyuan, Zhejiang Province of China, and dried at room temperature. Identity of *G. frondosa* was confirmed by Professor Changwen Ye (Edible Fungus Research Center, Zhejiang, China). 3‐(4,5‐Dimethylthiazol‐2‐yl)‐2,5‐diphenyltetrazolium bromide (MTT), cyclophosphamide (CTX), concanavalin A (ConA), lipopolysaccharide (LPS), and dimethyl sulfoxide (DMSO) were obtained from Sigma Chemical Co. All solvents/chemicals used were of analytical grade.

### 2.2 Animals

Balb/c strain mice (6 ~ 8 weeks old, 20 ± 2 g) were provided by the Comparative Medicine Center of Yangzhou University, China (the license number SCXK [SU] 2012–0004). The animals were acclimatized for 1 week before the experiment. During the experiment, the mice were housed under controlled environmental conditions of temperature (25 ± 2°C) with a normal day/night cycle and humidity (55 ~ 60%), and maintained on a basal diet and water ad libitum. All animal experiments were performed in accordance with the Code of Ethics of the World Medical Association and approved by the Ethics Committee of Yangzhou University.

### 2.3 Extraction and purification of *G. frondosa* polysaccharide

The crude polysaccharides were extracted from the fruiting bodies of *G. frondosa* using a method reported before (Li et al., 2018). Briefly, dry *G. frondosa* (40 g) was extracted with 1200 ml double‐distilled water at 100°C for 3 h, and the extraction process was repeated for three times. After centrifugation, the supernatants were combined and concentrated using a rotary evaporator. Then, four volumes of ethyl alcohol (EtOH) were added and the mixture was stored at 4°C overnight to precipitate polysaccharides.

The crude polysaccharides were purified by trichloroacetic acid method and column chromatography of DEAE‐52 cellulose. The precipitates were redissolved in water and placed in an ice bath, followed by a slow addition of 15% trichloroacetic acid until the pH reached 2.0 ~ 3.0. After remaining for 4 h, the supernatants were collected, centrifuged, and adjusted pH to 7.0 with 1 M NaOH. The solution was extensively dialyzed for 72 h (MWCO 3500 Da), and lyophilized to obtain the crude polysaccharides (GFP). GFP was redissolved and subjected to a DEAE‐52 cellulose column (1.6 cm × 50 cm), followed by a stepwise elution using an increasing concentration of NaCl (0, 0.05, 0.10, 0.15, and 0.20 M) at a flow rate of 1.0 ml/min. Fractions were collected and the sugar profile was monitored using the phenol‐sulfuric acid method. Fractions with the highest yield (0.05 M NaCl elution) were combined, concentrated, and lyophilized, generating the purified polysaccharide (GFP2).

### 2.4 Selenylation modification of GFP2

*Grifola frondosa* polysaccharide was selenylated using the HNO_3_‐Na_2_SeO_3_ method with minor modifications (Wang et al., 2016). Briefly, the purified GFP2 (30 mg) was dissolved in 0.7% HNO_3_ and stirred at room temperature for 10 h. Na_2_SeO_3_ of 24 mg and BaCl_2_ of 40 mg were added and reacted at 70°C for 6 h. After the reaction, the mixture was cooled to room temperature and the pH was adjusted to 7.0 ~ 8.0. Na_2_SO_4_ of 40 mg was added to remove the Ba^2+^. The supernatant was collected and dialyzed (MWCO 3000 Da) using distilled water until the reaction solution became colorless when detected by ascorbic acid method (Zhang, Zhang, et al., 2021). The resulting solution was concentrated, precipitated with EtOH, and freeze dried to obtain selenized *G. frondosa* polysaccharides (SeGFP2).

The selenium analysis was performed by atomic fluorescence spectrophotometry (AFS‐9950, Haiguang Analytical Co.) as reported by Li et al. (2017) and Gao et al. (2016). Briefly, the concentrations of Se standard solution were set at 0, 2, 4, 8, 16, 32 μg/L. The instrument automatically diluted, detected the fluorescence intensity, and then drew the standard curve. The regression equation was Y (fluorescence intensity) = 53.43X (Se concentration, μg/L)‐12.86 (*R* ^2^ = .9998). The GFP2 was digested with HClO_4_‐HNO_3_ (1:4) mixed solution for 12 h at 4°C, then heated at 180 ~ 190°C until it became clear accompanied with white smoke, concentrated to 1 ~ 2 ml. Six M HCl was added, heated, and the solution was concentrated to 1 ~ 2 ml, and then cooled and diluted into 10 ml with 6 M HCl. After detection, its Se content was calculated according to the regression equation. The Se yield was obtained by AFS over the weight of lyophilized extracts. All measurements were performed in triplicate.

### 2.5 FT‐IR spectroscopy

The FT‐IR spectrum (4000–500 cm^‐1^) was obtained using a NEXUS 670 FT‐IR spectrophotometer. Two milligram of SeGFP2 was completely mixed with 200 mg of KBr and pressed into flakes. Single‐beam spectra were collected against that of the background reference and converted to the absorbance.

### 2.6 Monosaccharide composition analysis

For GC analysis, SeGFP2 (5 mg) was hydrolyzed with 3 M H_2_SO_4_ at 110°C for 8 h. After totally removing the excess H_2_SO_4_, the resultant monosaccharides were converted into alditol acetates as described before (Li et al., 2018), and then analyzed by GC.

### 2.7 Methylation and GC‐MS analysis

The glycosidic linkage analysis of SeGFP2 was carried out using the methylation method (Wang et al., 2017). Specifically, SeGFP2 (5.0 mg) was dissolved in anhydrous DMSO with a nitrogen inlet. Dried NaOH (100 mg) was added and the mixture was stirred for 1 h. CH_3_I of 1.0 ml was added and the mixture was incubated in darkness for 4 h. The reaction mixture was extracted with chloroform, then the organic phase was washed with distilled water and dried under vacuum. After being methylated several times, the methylated SeGFP2 was confirmed by FT‐IR. The methylated SeGFP2 was hydrolyzed with 85% formic acid at 100°C for 6 h and 2 M trifluoroacetic acid (TFA) at 100°C for 6 h, and then reduced with NaBH_4_ and neutralized with acetic acid. The sample was acetylated by a procedure as mentioned in *Section* *2.6*. Subsequently, the partially *O*‐methylated alditol acetates (PMAAs) were detected by a GC‐MS (6890N/5975B GC‐MS, Agilent Co.) and the methylated SeGFP2 linkages were obtained by the retention time and fragmentation pattern.

### 2.8 HPSEC‐MALLS‐RI analysis

A high‐performance size exclusion chromatograph (HPSEC) coupled with a multiangle laser light scattering (MALLS) photometer (DAWN HELEOS 8, Wyatt Technology Co.) and a refractive index (RI) detector (2414 HPLC, Agilent) was used. Injection volume was 100 μl. Samples with a concentration of 5.0 mg/ml were filtered through 0.22‐μm syringe filters before injection. A TSK G‐6000PWXL column linked with a TSK G‐4000PWXL column (30 cm × 7.8 mm i.d., TOSOH) was used at 633 nm and 35°C. The eluents were 0.15 M NaNO_3_ with 0.05 M NaH_2_PO_4_ at 0.5 ml/min. Astra version 6.1.1 wyatt software (Wyatt Technology Co.) was used for the data acquisition and analysis.

### 2.9 Colorimetric determination with Congo red

The helix coil transition or random coils conformation of SeGFP2 was determined by Congo red test. Usually, SeGFP2 (5 mg) was dissolved in water and then mixed with 80 μM Congo red dye. One M NaOH was dropwise added into the mixture to achieve 0 ~ 0.5 M final concentrations, and the absorbance (A) was recorded on an ultraviolet‐visible spectrophotometer (UV‐2450, Shimadzu Co.). The optical rotation of mixture alkaline solution without polysaccharides was used as the reference.

### 2.10 Microscopic analysis

The atomic force microscopy (AFM) was employed to observe the molecular morphology of SeGFP2. SeGFP2 (10.0 µg/ml) was dispersed in water and filtered through a 0.45 μm syringe filter, and 10.0 µl of sample solution was deposited onto the freshly cleaved mica and then air dried for 4 ~ 8 h at room temperature. The sample was examined in the tapping mode with a Multimode 8 (Bruker) in air. The Nanoscope software (Build R3Sr6.^104^281, Bruker Corporation) was performed for image manipulation.

### 2.11 Splenocyte proliferation assay

Lymphocyte proliferation was assessed by an MTT‐based colorimetric assay. Balb/c mice (6 ~ 8 weeks old, 20 ± 2 g) were sacrificed via cervical dislocation. Spleens were aseptically removed and placed in cold RPMI‐1640 medium under aseptic conditions, then gently homogenized, passed through a 40 μm nylon cell strainer to generate single‐cell suspensions. After removal of erythrocytes from the cell mixture, the cells were washed twice and suspended in RPMI 1640 medium supplemented with 10% fetal bovine serum, adjusted to a final density of 5 × 10^6^ cells/ml. Aliquots of 100 μl of splenocytes (5 × 10^6^ cells/ml) were placed in a 96‐well plate with or without ConA (10 μg/ml) or LPS (20 μg/ml). Samples of different concentrations (0, 25, 50, or 100 μg/ml) were added to each well and the plate was incubated at 37°C in a humidified 5% CO_2_ incubator for 72 h. Twenty microliters of MTT (5 mg/ml) was added per well and incubated for 4 h, followed by the addition of DMSO (150 μl/well). The absorbance at 570 nm was measured using a microplate reader (BioTek Synergy H4).

### 2.12 Antioxidant activity analysis

The in vitro antioxidant activities of SeGFP2 and GFP2 were evaluated using the free radical scavenging activities and ferrous ion‐chelating abilities. The DPPH radical scavenging activity was measured according to the method described by Li et al. (2017). Briefly, 2 ml of aqueous aliquots (0, 200, 400, 600, 800, 1000, 1500, and 2000 μg/ml) was mixed with 2.0 ml DPPH solution (0.2 mM in EtOH). The mixture was vortexed intensely, and then allowed to settle for 30 min under dark condition. The DPPH radical scavenging effect was evaluated according to the following equation: Scavenging rate (%) = [(A_B_‐A_S_)/A_B_] ×100%, where A_B_ and A_S_ separately represent the absorbance of blank and test sample. Ascorbic acid was used as a positive control to validate the assay.

The ABTS^+^ radical scavenging activity was measured following a modified scheme based on Jeddou et al. (2016). The ABTS^+^ solution was produced by reacting 1.82 mM ABTS^+^ with 1.07 mM potassium persulfate. Then, the mixture was left to settle for 24 h under dark condition. The ABTS^+^ solution was diluted with 0.15 M sodium phosphate‐buffered saline (pH 7.4) to an initial absorbance of 0.70 ± 0.02 (734 nm). Prior to the assay, 0.2 ml of sample solutions was added to 3.8 ml of diluted ABTS^+^ radical solution. The absorbance was measured at 734 nm after a 6‐min incubation. The ABTS^+^ radical scavenging activity was calculated using the following equation: Scavenging rate (%) = [(A_B_‐A_S_)/A_B_] ×100%, where A_S_ and A_B_ separately represent the absorbance values of ABTS^+^ solution with and without test sample. Ascorbic acid was used as a positive control.

The ferrous ion‐chelating ability was performed following the modified method described by Yuan et al. (2020). Briefly, the sample in different concentrations (0, 200, 400, 600, 800, 1000, 1500, and 2000 μg/ml) was mixed with 3.7 ml of distilled water, and then reacted with 0.1 ml FeSO_4_ (2.0 mM). After 0.2 ml of 5.0 mM ferrozine was added, the solution was mixed, and allowed to remain for 10 min at room temperature. The absorbance was determined at 562 nm. The chelating activity on ferrous ions was calculated using the following equation: Chelating ability (%) = [(A_B_‐A_S_)/A_B_] ×100%, where A_B_ and A_S_ separately represent the absorbance of blank and test sample. EDTA was co‐assayed as a positive control.

### 2.13 Statistical analysis

Data analysis was performed with SPSS software (version rel. 18.0, SPSS Inc.). Differences were considered statistically significant at *p* < .05.

## 3 RESULTS AND DISCUSSION

### 3.1 Extraction, purification, and general analysis of SeGFP2

A homogeneous polysaccharide fraction was purified by DEAE‐52 column and named GFP2 (Li et al., 2018). The UV spectrum of GFP2 exhibited a decreasing absorbance similar to that of most polysaccharides, a negative response to the Bradford test and no absorption peaks at 260 or 280 nm, indicating the absence of nucleic acids and proteins.

**FIGURE 1:**
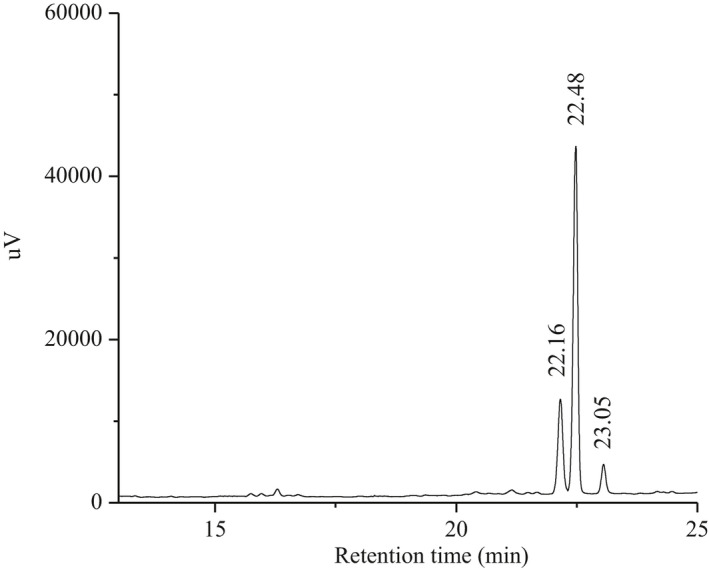
Gas chromatograms of the monosaccharide compositions of SeGFP2

The Se content of SeGFP2 was detected to be 445.39 μg/g. Organic Se fortification of this mushroom source could help to alleviate Se deficiency in the population of China, in addition, making more Se‐fortified food choices available. Similar Se content was also found in the selenized polysaccharide (SeASP_6_) from *Artemisia sphaerocephala* after the HNO_3_‐Na_2_SeO_3_ synthesis method (Wang et al., 2012). The alditol acetates of acid hydrolyzed SeGFP2 were analyzed by GC; in conclusion, it contained mannose, glucose, and galactose in a ratio of 3.5:11.8:1.0 (Figure 1). SeGFP2 was a heteropolysaccharide, in which D‐glucose was the dominant constituent. A higher proportion of glucose was indicated in SeGFP2 compared with the previous reported study on *G. frondosa* polysaccharide, which consisted of glucose (64.4%), galactose (25.7%), and mannose (9.9%) (Xu et al., 2010).

### 3.2 FT‐IR spectra

**FIGURE 2:**
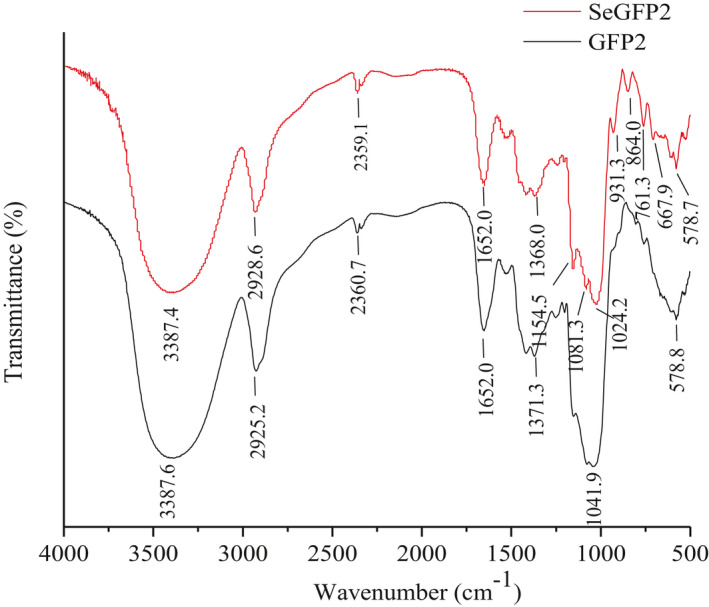
FT‐IR spectra of SeGFP2 and GFP2

The characteristic absorptions of SeGFP2 and GFP2 were performed using FT‐IR spectra (4000 ~ 500 cm^‐1^) (Figure 2). In the spectrum of GFP2, there were four characteristic absorption peaks at 3387.6 cm^‐1^, 2925.2 cm^‐1^, 1652.0 cm^‐1^, and 1371.3 cm^‐1^, respectively, being the stretching vibration absorption peaks of O‐H (3500 ~ 3300 cm^−1^), C‐H (3000 ~ 2800 cm^−1^), C = O (1700 ~ 1500 cm^−1^), and C‐O (1400 ~ 1000 cm^−1^). The absorption band in the region of 1000 ~ 1200 cm^‐1^ was dominated by ring vibration overlapped with the C‐O‐C glycosidic band vibration and C‐OH stretching vibration of pyranose in polysaccharides. The lack of carbonyl bands around 1750 cm^‐1^ indicated the absence of uronic acids in GFP2 and SeGFP2 (Liu et al., 2013).

While most peaks were also shown in the spectrum of SeGFP2, two new absorption peaks appeared at 667.9 cm^‐1^ and 1024.2 cm^‐1^, respectively, assigned to the Se‐O‐C stretching vibration (ν, 700 ~ 600 cm^‐1^) and the O‐Se‐O stretching vibration (ν_as_, 1040 ~ 1010 cm^‐1^) (Hou et al., 2016). This signified that Se had been combined to the polysaccharide molecule. The absorption band at 864.0 cm^‐1^ was ascribed to β‐type glycosidic linkages (Zhang, Zhou, et al., 2016). Previous studies indicated that active polysaccharides in those mushrooms appeared to have potential immunoregulatory activity, primarily due to the polysaccharides with β‐glucan structures (Xu et al., 2012).

### 3.3 Glycosidic linkages

**FIGURE 3:**
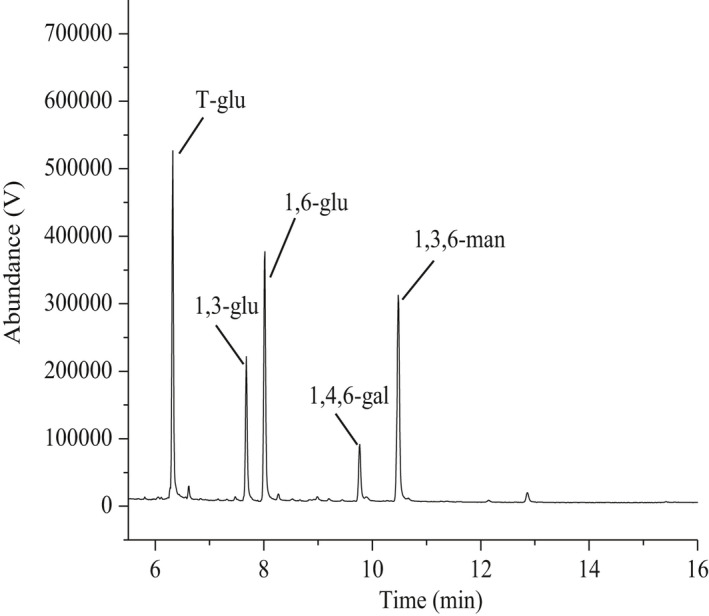
The total ion chromatograms from methylation analysis of SeGFP2

As summarized in Table 1 and Figure 3, the results showed the presences of five types of linkages: 2,3,4,6‐Me_4_‐Glc*p*, 2,4,6‐Me_3_‐Glc*p*, 2,3,4‐Me_3_‐Glc*p*, 2,3‐Me_2_‐Gal*p*, and 2,4‐Me_2_‐Man*p* in a mole ratio of 5.3:2.4:4.2:1:3.7. It suggested that SeGFP2 might be proposed as a branched polysaccharide consisting of 1,3‐linked‐D‐Glc*p*, 1,6‐linked‐D‐Glc*p*, 1,4,6‐linked‐D‐Gal*p*, and 1,3,6‐linked‐D‐Man*p* units. The mole ratio was nearly in agreement with the monosaccharide composition. The degree of branching (DB) was 60.3% based on the calculation method reported by Chen et al. (2015).

**TABLE 1:** GC‐MS data for methylation analysis of SeGFP2

Methylated sugar	*t_R_* (min)^b^	Linkage pattern	MS (m/z)	Molar ratio
2,3,4,6‐Me_4_‐Glc*p* ^a^	6.32	T‐Glc*p*	43,45,71,87,101,113,117,129,145,161,205	5.3
2,4,6‐Me_3_‐Glc*p*	7.68	1,3‐linked‐Glc*p*	43,58,71,87,101,117,129,161,233	2.4
2,3,4‐Me_3_‐Glc*p*	8.02	1,6‐linked‐Glc*p*	43,87,99,101,117,129,161,189,233	4.2
2,3‐Me_2_‐Gal*p*	9.90	1,4,6‐linked‐Gal*p*	43,85,99,101,117,127,142,161,261	1
2,4‐Me_2_‐Man*p*	10.48	1,3,6‐linked‐Man*p*	43,71,87,101,117,129,189,233	3.7

^a^ 1,5‐di‐*O*‐acetyl‐2,3,4,6‐tetra‐*O*‐methyl‐gluctiol.

^b^ *t_R_*, Relative retention time.

### 3.4 Molecular weight and chain conformation

**FIGURE 4:**
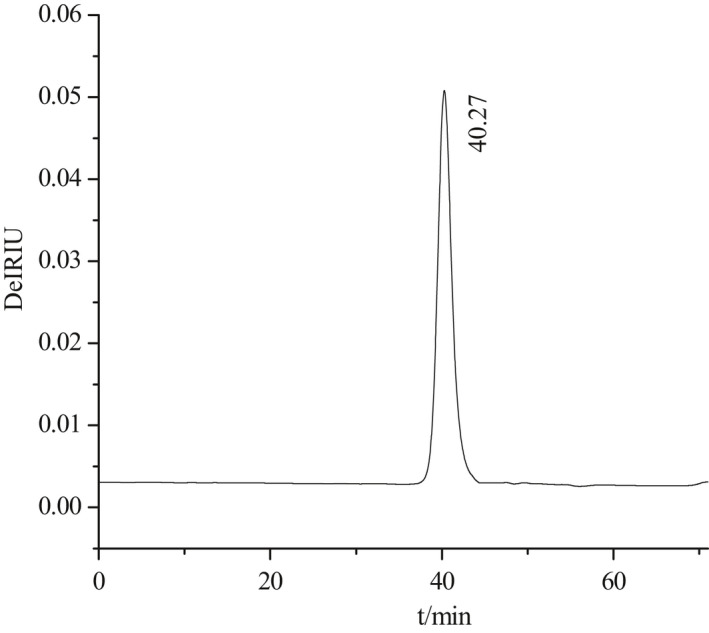
Light scattering signals of SEC chromatogram of SeGFP2

High‐performance size exclusion chromatography–multiangle laser light scattering–refractive index detector was used as an efficient method to determine the molecular conformation and related parameters of the polysaccharide in dilute polymer solution. As shown in Figure 4, a single symmetrical peak was observed in the HPSEC chromatogram, and indicated that SeGFP2 was a homogeneous polysaccharide with the weight‐average Mw of 2.12 × 10^4^ Da. The polydispersity index M_w_/M_n_ was 1.068, suggesting a polydisperse polymer in SeGFP2. The radius of gyration (R*_g_*) is known as the distance between the mass center and the segment. The value of Z‐average R_z_ was determined to be 13.5 nm. For a given polymer solution, the gradient value (ν) may provide additional insights into macromolecule conformation and architecture. Usually, the ν values of 0.33, 0.50 ~ 0.60, and 1.0 separately exhibit the sphere, random coil, and rigid rod of the polymer (Zhao et al., 2014). The ν value of SeGFP2 was 0.40, which suggested that SeGFP2 molecules in an aqueous solution might be in a state between spheres and random coils.

**FIGURE 5:**
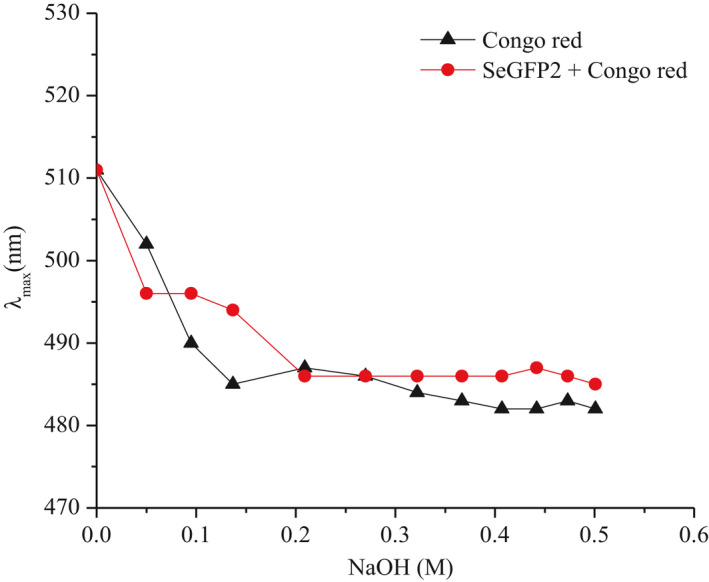
Absorption spectra of Congo red (control) and Congo red with SeGFP2 at various concentrations of NaOH

Congo red test was performed to detect the triple helix or random coils structure of polysaccharide chains in an aqueous alkaline solution. Figure 5 shows the change of maximum absorbance (λ_max_) of SeGFP2‐Congo red complex at a NaOH concentration (0 ~ 0.5 M). Obviously, the addition of SeGFP2 to the Congo red solution did not cause any notable changes in λ_max_ from 480 nm to 520 nm compared with that of Congo red alone, suggesting that SeGFP2 chains existed as random coils instead of helical structure. Furthermore, our results are consistent with the other bioactive polysaccharides, which also exhibited as random coils (Lavi et al., 2006).

### 3.5 Molecular morphology

**FIGURE 6:**
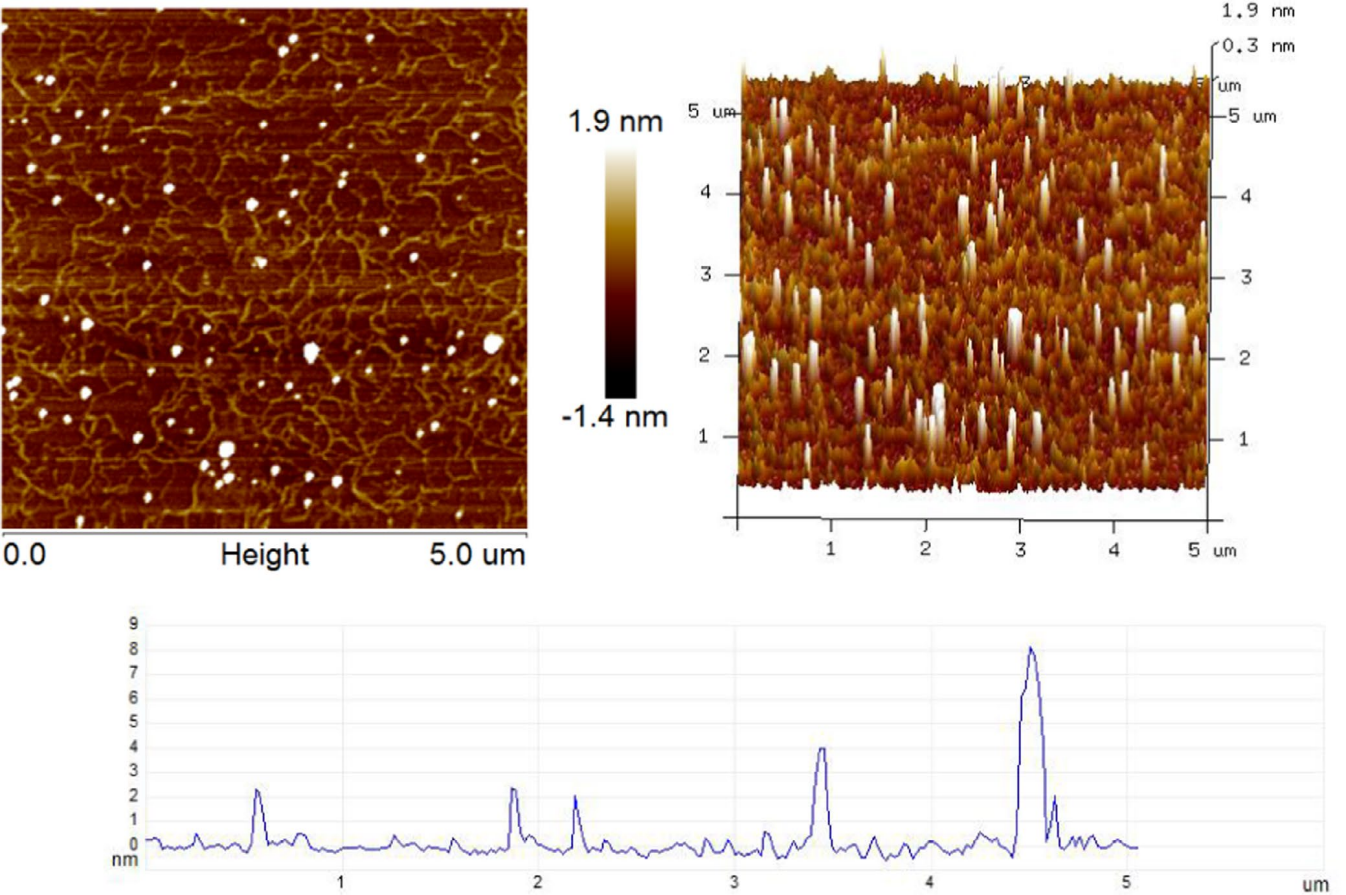
AFM images of SeGFP2 obtained under tapping mode using a Multimode 8 instrument (Bruker)

Generally, AFM is useful for observing the surface and topography of each sample (Kong et al., 2015). AFM images of SeGFP2 were provided in Figure 6. SeGFP2 appeared as worm‐like chains and the molecular chains branched and entangled with each other at a concentration of 10.0 μg/ml. The height of all observed chains was around 0.3 ~ 8.1 nm, which is consistent with the thickness of multiple molecular chains (Liu et al., 2013). The molecular aggregation was ascribed to the ‐OH groups of SeGFP2, which provided inter/intra‐molecular interactions with each other or water molecule (Kong et al., 2015).

### 3.6 Lymphocyte proliferation

**FIGURE 7:**
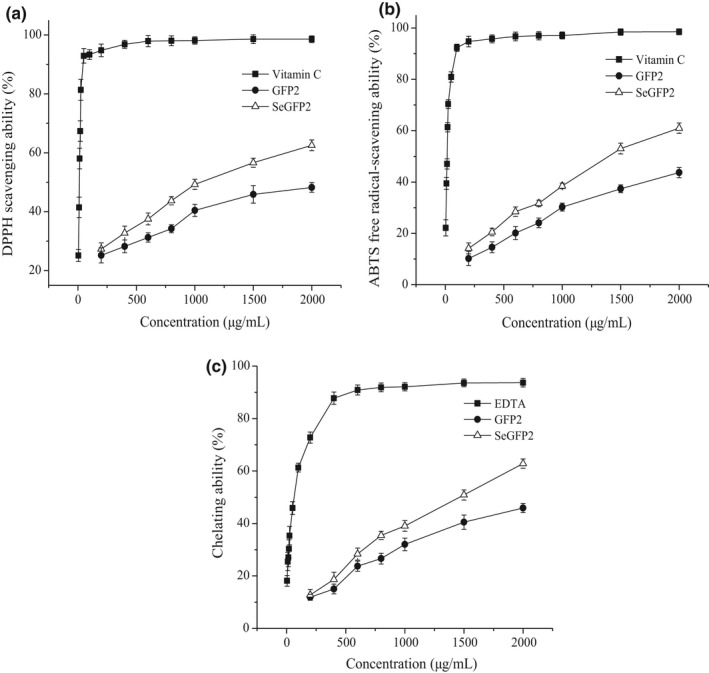
Evaluation of in vitro antioxidant activity of SeGFP2. (a) DPPH radical scavenging activity, (b) ABTS^+^ radical scavenging activity, and (c) chelating effect on ferrous ions

Many fungal polysaccharides can activate T lymphocyte and B lymphocyte to show their effects on the immune system (Liu et al., 2017). Lymphocyte proliferation is the most direct indicator of immunoactivation. Usually, lymphocytes induced by ConA or LPS are, respectively, used to evaluate T‐ or B‐lymphocyte activity (Liu et al., 2017). As shown in Figure 7a, the lymphocyte proliferation rate in SeGFP2 or GFP2 group was significantly (*p* < .05) higher than that of the control group. SeGFP2 groups at 25, 50, and 100 μg/ml were significantly (*p* < .01) higher than corresponding ConA control group (Figure 7b). Synergistic effect was observed between polysaccharide and LPS, especially at the medium and high concentrations (Figure 7c). The T‐lymphocyte proliferation effects of two polysaccharides combined with ConA were presented in a concentration‐dependent manner. With the synergistic effect of SeGFP2 and LPS, B‐lymphocyte proliferation exhibited a dose‐dependent trend. The results confirmed that two polysaccharides at suitable concentrations could significantly induce the lymphocyte proliferation, synergistically with ConA or LPS. SeGFP2 treatment as an adjuvant could significantly promote T‐ or B‐lymphocyte proliferation combining with ConA or LPS, and the enhancement was higher than GFP2.

Immunostimulation itself is regarded as one of the important strategies to improve the host defense mechanism in humans as well as cancer patients. Various experiments proved that polysaccharides from mushrooms could enhance the host immune system by stimulating T cells, B cells, natural killer cells, and macrophage cells (Liu et al., 2017; Xu et al., 2012). *Grifola frondosa* polysaccharides were reported to show immunostimulatory activities, such as the improvement of RAW264.7 cells proliferation and the macrophage‐activating capability (Meng et al., 2017). In fact, immunostimulatory activities of polysaccharides depend on the structural information such as monosaccharide constituent, glycosidic linkage, molecular weight, and function groups. It was reported that β‐glucans from mushrooms, especially β‐1,3‐ and β‐1,6‐linkages, were important for increasing cell immune activity (Liu et al., 2017; Xu et al., 2012). Sun et al. (2012) reported that a relatively low molecular weight of the polysaccharide was desired for its immunostimulatory activity. The current study firstly demonstrated that SeGFP2, with a M_w_ of 2.12 × 10^4^ Da, consisted of 1,3‐linked‐D‐Glc*p*, 1,6‐linked‐D‐Glc*p*, 1,4,6‐linked‐D‐Gal*p,* and 1,3,6‐linked‐D‐Man*p* units. Our results are in agreement with these discussions.

Se‐polysaccharides were immune response regulators as reported in several studies (Gao et al., 2016). Haibo et al. (2016) reported that the selenylation modification of *Chuanminshen violaceum* polysaccharides (sCVPS) was obtained using HNO_3_‐Na_2_SeO_3_ method. The selenylation of CVPS could significantly increase the immunoregulatory activity both in vitro and in vivo, thus representing a powerful adjuvant for vaccine design. It was elucidated that Se alone could improve the abnormal levels of cytokines and oxidative damages in chicken spleen, thus ameliorating the injury induced by heat stress. The combination of Se and polysaccharides induced a higher immune function (Zhang, Gao, et al., 2021). Thus, these structural features may be responsible for the higher lymphocyte proliferation activity of SeGFP2. Further studies should be made to elucidate the immunostimulatory activity and its possible mechanism.

### 3.7 Antioxidant activity

**FIGURE 8:**
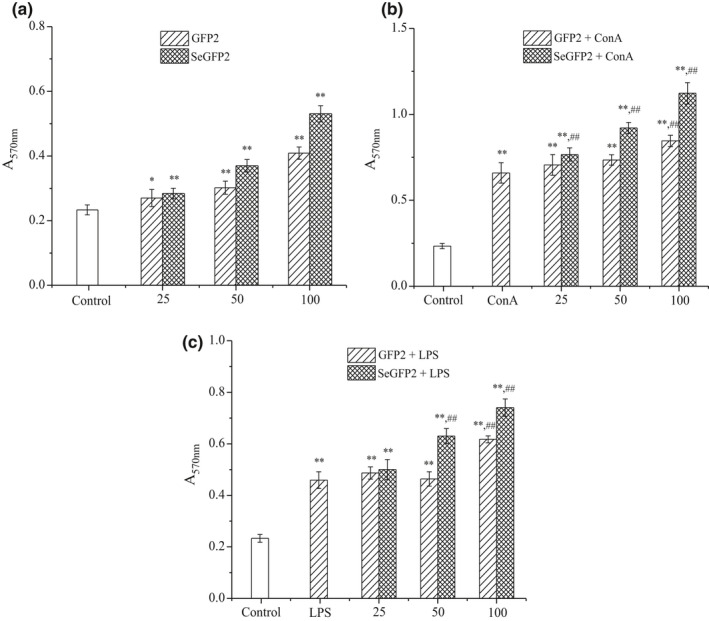
Effect of SeGFP2 and GFP2 with or without ConA or LPS on splenocyte proliferation. (a) Polysaccharides, values are given as means ± *SD*; *p < .05, **p < .01 vs. negative control; (b) Polysaccharides +ConA, values are given as means ± *SD*; *p < .05, **p < .01 vs. negative control; ^#^ *p* < .05, ^##^ *p* < .01 vs. ConA; (c) Polysaccharides +LPS, values are given as means ± *SD*; *p < .05, **p < .01 vs. negative control; ^#^ *p* < .05, ^##^ *p* < .01 vs. LPS

As illustrated in Figure 8a, the DPPH radical scavenging abilities of SeGFP2 and GFP2 were both concentration dependent (200 ~ 2000 μg/ml). The DPPH radical scavenging ability ranged from 25.18% to 48.24% for GFP2, while from 27.31% to 62.53% for SeGFP2. At the concentration of 2000 μg/ml, the inhibition percentage of SeGFP2 was 62.53% ± 1.85%, which was higher than that of GFP2 (48.24% ± 1.66%).

The ABTS^+^ radical has been widely used to measure the total antioxidant activity of single compounds or complex mixtures (Jeddou et al., 2016). As shown in Figure 8b, the effect of ABTS^+^ radical scavenging was presented in a concentration‐dependent manner (200 ~ 2000 μg/ml). The ABTS^+^ radical scavenging rate of SeGFP2 was 61.01% ± 2% at 2000 μg/ml, nearly 1.40‐fold higher than that of GFP2 (43.67% ± 1.97%). It suggested that SeGFP2 showed a noticeable ability on the scavenging of ABTS^+^ radicals, especially at a high concentration.

The chelating agents, which form bonds with metals, are effective as secondary antioxidants for the redox potential reduction, thus stabilizing the oxidized form of the metal ions (Yuan et al., 2020). Both SeGFP2 and GFP2 showed antioxidant activities, and the Fe^2+^‐chelating ability was, respectively, 62.83% and 45.9% at a dose of 2000 μg/ml, lower than that of EDTA (Figure 8c). Basically, the chelating ability of SeGFP2 was a little superior to that of GFP2 under other six concentrations. As described by Yuan et al. (2020), the chelating ability of polysaccharides on Fe^2+^ might affect the other radical scavenging activities to protect the organism against oxidative damage. Since Fe^2+^ is the most effective pro‐oxidant in food system, the high Fe^2+^‐chelating abilities of polysaccharides from *G. frondosa* fruit bodies would be somewhat beneficial in the antioxidation.

It was reported that the antioxidant ability of polysaccharides was due to their hydrogen‐donating effects. The element Se in SeGFP2 could activate the hydrogen atom of the anomeric carbon (Turło et al., 2010; Zhang, Lu, et al., 2016). The higher irritation ability of the group led the hydrogen atom‐donating ability stronger. This suggested that selenylation modification could enhance the in vitro antioxidant activity. In fact, a relatively low molecular weight of polysaccharides was highly desired for the antioxidant ability (Zhao et al., 2014). SeGFP2 with a M_W_ of 2.12 × 10^4^ Da exhibited a stronger antioxidant ability than Se‐GFP‐22 (4.13 × 10^6^ Da), which was reported in our previous study (Li et al., 2017). The antioxidant ability of the polysaccharides also strongly depended on the type of sugar monomers, the linkage pattern of the backbone, and the degree of branching.

## 4 CONCLUSION

In this study, *G. frondosa* polysaccharides were extracted and purified by anion‐exchange chromatography, and modified in selenylation by HNO_3_‐Na_2_SeO_3_ method for the first time. The lymphocyte proliferation and antioxidant activities of SeGFP2 were also evaluated taking GFP2 as control. A typical absorption of selenium ester in SeGFP2 molecule was observed in FT‐IR. SeGFP2 was composed of mannose, glucose, and galactose in a ratio of 3.5:11.8:1.0. SeGFP2 exhibited as a branched polysaccharide consisted of 1,3‐D‐Glc*p*, 1,6‐D‐Glc*p*, 1,4,6‐D‐Gal*p,* and 1,3,6‐D‐Man*p*. The current study firstly demonstrated that SeGFP2 (M_w_ 2.12 × 10^4^ Da) possessed synergistic stimulation effects for T‐ or B‐lymphocyte proliferation combining with ConA or LPS. The lymphocyte proliferation in SeGFP2 group was higher than in the GFP2 group. The in vitro antioxidant activities of SeGFP2 were more potent than GFP2. In summary, selenylation modification could enhance the antioxidant and immunostimulatory activities of GFP2, which might be related to its moderate molecular weight, Se content, monosaccharide constituent, glycosidic linkage, and function groups. Overall, SeGFP2 could be considered as a potential immunomodulatory agent with antioxidant activity or a dietary Se‐supplement. Further investigations of the detailed mechanisms underlying the immunostimulatory activity of SeGFP2 are still necessary.

## CONFLICT OF INTEREST

There are no conflicts to declare.

## ETHICAL APPROVAL

Animal experiments were performed in accordance with the code of ethics of the World Medical Association and approved by the Ethics Committee of Yangzhou University.

## Data Availability

The data that support the findings of this study are available from the corresponding author upon reasonable request.
